# Intravenous leiomyomatosis presenting as Budd–Chiari syndrome: a case report and literature review

**DOI:** 10.1186/s13023-025-03556-z

**Published:** 2025-02-04

**Authors:** Jingwen Gan, Xiao Ma, Jiang Shao, Jinhui Wang, Dongyan Cao

**Affiliations:** 1 National Clinical Research Center for Obstetric and Gynecologic Diseases, Department of Obstetrics and Gynecology, Peking Union Medical College Hospital (Dongdan campus), Chinese Academy of Medical Sciences and Peking Union Medical College, No.1 Shuaifuyuan Wangfujing Dongcheng District, Beijing, 100730 China; 2 Department of Vascular Surgery, Peking Union Medical College Hospital, Chinese Academy of Medical Sciences and Peking Union Medical College, Beijing, China

**Keywords:** Intravenous leiomyomatosis, Budd–Chiari syndrome, Hepatic veins outflow obstruction, Abdominal distention, Lower extremity edema

## Abstract

**Background:**

Budd–Chiari syndrome (BCS) caused by intravenous leiomyomatosis (IVL) is rare. Further reports and thorough evaluation are needed to identify and manage this disease.

**Method:**

We described the case of a 49-year-old lady, exhibiting features of BCS secondary to IVL, and reviewed three other previous cases of BCS caused by IVL.

**Results:**

The mean onset age of these four patients was 54.8 years. All but one (Patient No. 2) had a history of myoma, myomectomy, or hysterectomy. Abdominal pain, bloating or increasing abdominal circumference, and bilateral lower extremity edema were common symptoms. The establishment of clinical diagnoses of IVL and BCS mainly depends on clinical presentations and imaging, such as ultrasonography, computed tomography, and magnetic resonance imaging. Surgical intervention to alleviate the hepatic veins outflow obstruction is the most important treatment.

**Conclusions:**

BCS caused by IVL should be considered when the inferior vena cava and right atrium lesions were detected in a patient with characteristics of BCS and a history of uterine myoma or hysterectomy. Complete tumor resection is the only curative treatment and should be performed as soon as possible.

## Introduction

Intravenous leiomyomatosis (IVL) is a rare variant of leiomyoma that may originate from either uterine leiomyoma with extensive vascular invasion or from the venous walls [1]. Reported cases indicate an onset age ranging from 21 to 80 years old [2], with IVL predominantly occurring in premenopausal women during the fourth and fifth decade [3]. The majority of patients have a surgical history of myomectomy and hysterectomy. The tumor can maliciously invade blood vessels and enter the systemic venous circulation via two different pathways: the ovarian vein to renal veins and inferior vena cava (IVC, bypassing the iliac veins), or the parametrial uterine vein to iliac veins and IVC, or both [1, 4]. Once the tumor extends into the IVC, it will progressively grow into the right atrium, right ventricle, pulmonary artery, and even subclavian vein [5]. Generally, the clinical presentations of IVL are diverse and nonspecific but predominantly depend on its location, size, disease progression, and extent of involvement. When the tumor invades the iliac veins and IVC, patients often present with lower extremity edema, and intravenous space-occupying lesions mimicking thrombosis. In extremely rare cases, IVL with extension into the IVC can lead to Budd–Chiari Syndrome (BCS). BCS is a rare condition caused by hepatic venous outflow obstruction involving one or more draining hepatic veins [6, 7]. To our knowledge, only three cases of BCS caused by IVL have been reported [6–8]. Herein, we report the fourth patient with IVL presenting as BCS. Combined our case with the previously reported cases, this article may provide a better understanding of BCS caused by IVL.

## Case presentation

A 49-year-old woman, para 2, presented to our hospital with an 11-month history of bilateral leg edema accompanied by abdominal distension. She denied any gynecologic symptoms and reported artificial menopause after a total abdominal hysterectomy (TAH) for a broad ligament myoma 2 years before. Her surgical history included a myomectomy surrounding the bladder 1 year ago and left lower limb varicose veins. On presentation, her vital signs were stable, with a blood pressure of approximately 100/80 mmHg, heart rate of about 60 beats per minute, and a respiratory rate of 18 breaths per minute. Abdominal physical examination indicated no obvious abnormity except hepatomegaly and varicose veins in the abdominal wall and the right lower back. Tympany on percussion of the abdomen was shown and shifting dullness was negative. She had severe pitting edema (+++) over both legs. Moreover, varicosis of the great saphenous vein was noted on the left lower limb.

**Fig. 1:**
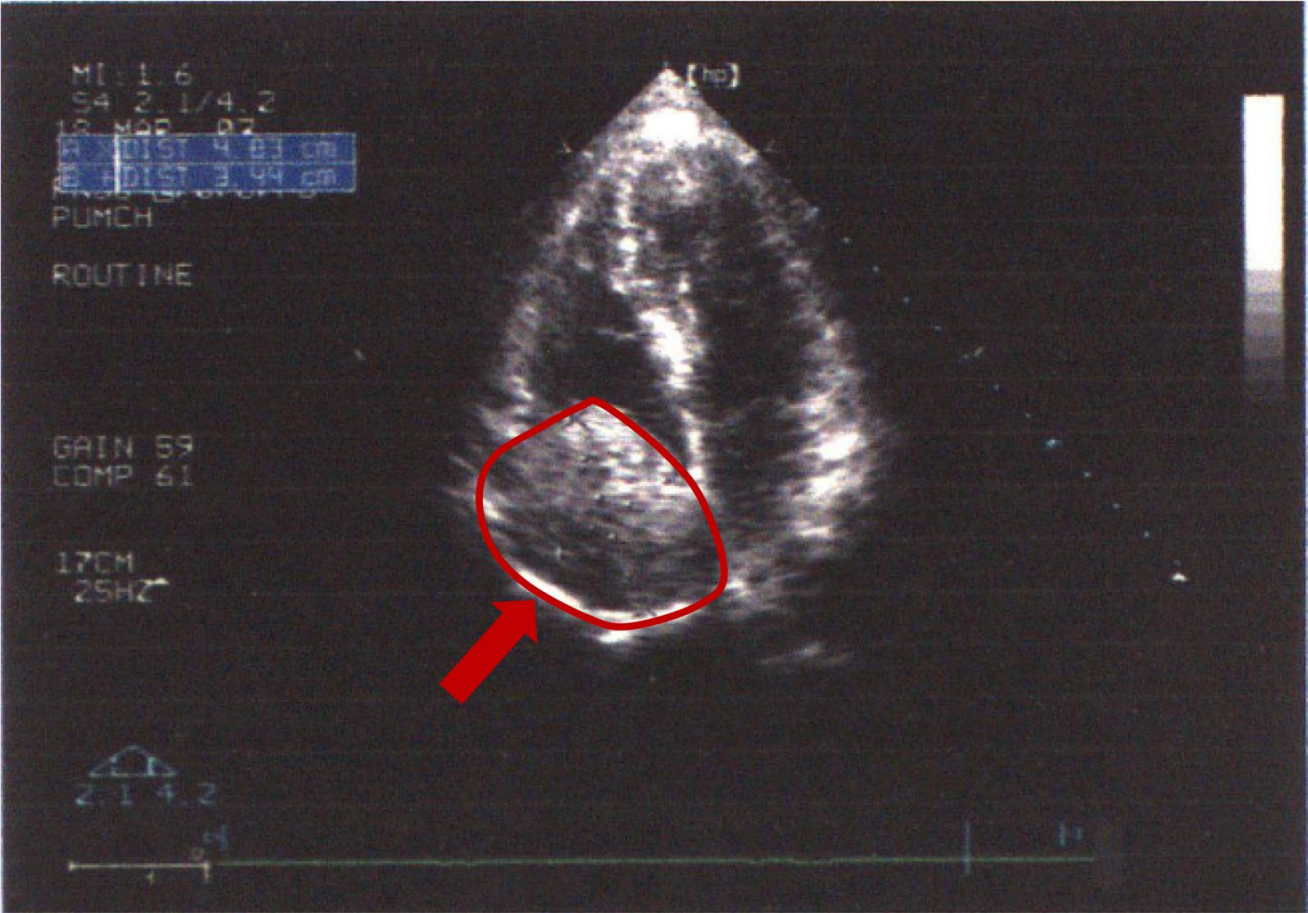
Echocardiogram showing an intracardiac tumor located in the right atrium (red arrow)

Pelvic ultrasonography revealed a 10.9 × 7.3 cm lobulated mass with isoechoic texture located in the pelvis. Vascular ultrasound examination showed the IVC and iliac venous system were occupied by a heterogeneous, avascular mass with hypoechoic regions, which was initially presumed to be a thrombosis. The diameter of the hepatic retro, middle, and lower segments of the IVC were 3.5 cm, 2.8 cm, and 1.7 cm, respectively. The bilateral common iliac veins were 1.0 cm wide. Abdominal ultrasound could greatly show hepatomegaly with heterogeneous enhancement and the occlusion of the hepatic venous reflux, while upper gastrointestinal radiography shows no abnormality. Abdominal and pelvic computed tomography angiography (CTA) evaluation was performed to further define intravenous lesions. Hepatic parenchymal nodules with hyperemia and septation enhancement were noted. CTA also showed a right common iliac vein—IVC—right atrium (RA) filling defect, highly indicative of tumor emboli; an irregular, soft-tissue, space-occupying lesion was found in the right pelvic cavity suggestive of neoplasm. Echocardiography detected that the entire RA was occupied by an oval shape, dense mass measuring 4.8 × 3.4 cm (Fig. 1). Laboratory tests showed elevated serum total bilirubin (TBIL, 2.39 mg/dl, normal range: 0.3–1.3 mg/dl), direct bilirubin (DBIL, 1.14 mg/dl, normal range: 0.01–0.5 mg/dl), total bile acid (TBA, 29.1 μmol/L, normal range: 0.1–10.0 μmol/L), and gamma-glutamyltransferase (GGT, 136 U/L, normal range: 10–50 U/L); whereas alanine aminotransferase (ALT), albumin (ALB), aspartate aminotransferase (AST), and alkaline phosphatase (ALP) were all within normal limits. The serum tumor markers including carbohydrate antigen- (CA-)199, CA-242, CA-50, and carcinoma embryonic antigen were also within normal ranges. Other routine investigations were unremarkable.

The pathologic specimens of her past surgery were reviewed again by the pathologists in the Department of Pathology at our institution, which was consistent with the diagnosis of leiomyoma with abundant tumor cells. A tentative diagnosis of BCS as a complication of IVL was established following a comprehensive evaluation. Subsequently, a multidisciplinary discussion was arranged, which comprised the Department of Obstetrics and Gynecology, Vascular Surgery, Urology, Radiology, Anesthesiology, Blood Transfusion, and Intensive Care Unit. The gynecologists and vascular surgeons developed the surgical strategy and performed the surgery together. To reduce surgical injury, the urologist placed double-J tubes under local anesthesia for the patient 1 day before the operation. In addition, radiologists reviewed images to evaluate the extent of the tumor invasion. Finally, the Department of Anesthesiology, Blood Transfusion, and Intensive Care Unit jointly participated in the perioperative management of the patient.

The gynecologists started the operation through the left side incision from the pubic union to 5 cm above the navel. On laparotomy, no ascites was found in the peritoneal cavity. After intraoperative exploration and adhesiolysis, the gynecologists found and resected two lymph nodes about 2 × 2 cm in size on each of bilateral iliac vessels. Both intraoperative frozen rapid pathology and postoperative pathology confirmed a diagnosis of lymph node reactive hyperplasia. Additionally, a massive varicose vein was identified on the right posterior peritoneum, which was about 4 × 5 cm in size and originating from the proper ligament of the right ovary. After careful examination, with no other tumors identified in the pelvis, the gynecologic surgeons removed the pelvic tumor and performed bilateral adnexectomy concurrently. Thereafter, the vascular surgeons extended the incision to the level of the xyphoid and observed the dilated IVC containing tumor thrombus. The IVC was mobilized and a tourniquet was applied to the IVC below the renal veins. Then, a 4 cm vertical incision was performed directly on the anterior wall of the IVC. The lumen of IVC was almost occupied by tumor thrombus with a diameter of 4 cm. The distal part of the tumor was freely removed through the venotomy; however, the proximal part of the tumor cannot be pulled downward out of the IVC. To prevent lacerations of the IVC, the distal part of the tumor was transected at the level of renal veins. Subsequently, the 4-cm incision in the IVC was extended to the level of the suprarenal IVC. The proximal portion of the tumor, measuring 16 cm in length and having a rat-tail shape, was then completely pulled downward out of the IVC. Finally, the common iliac veins were opened, revealing a few tumor remnants in the common and internal iliac veins, which were easily removed using hemostatic forceps. The IVC was sutured by continuous suture without complications. The blood loss volume was approximately 6000 ml. The autologous blood transfusion was applied intraoperatively. A total of 1200 ml autologous blood and 5 units of packed red cells were transfused.

Although radical surgical resection of pelvic tumor, venous thrombus, and cardiac tumor thrombus was initially planned, the resection of the cardiac tumor thrombus finally was not performed to prioritize the patient’s safety. Considering the surgery is time-consuming and traumatic, with significant blood loss, the patient was admitted to the ICU, where heparin was administered to prevent postoperative thrombosis due to the easy adjustment of the amount of heparin according to the patient's coagulation function. When the patient exhibits no significant risk of postoperative bleeding and the coagulation function is stable, we turned to low molecular weight heparin, which is more user-friendly. Final histopathology favored the diagnosis of BCS caused by IVL with intravenous extension. The patient’s clinical symptoms of abdominal distension and varicose veins, and severe edema of both lower limbs significantly improved postoperatively. Although the patient experienced jaundice, she was well-healed by adjustment to enteral nutrition, intravenous hepatoprotective treatment, and antimicrobial therapy after consulting with an infectious medicine specialist who considered surgical stress and long-term postoperative parenteral nutrition as the reasons of postoperative jaundice. All of serum indicators almost recovered to preoperative level when she discharged on day 37 postoperatively. To reduce the risk of tumor embolism caused by incomplete resection and the risk of postoperative venous thromboembolism, lifelong anticoagulation is recommended in the absence of contraindications. Therefore, the patient turned to warfarin when she was discharged.

Three and a half months post-surgery, space-occupying lesions were found in the IVC about 2 cm in size, excluding the residual tumor in the RA according to the ultrasound from local hospital. Eleven years postoperatively, the patient remains alive with the disease. Since then, she was lost to follow-up.

## Discussion

IVL is a rare histologically benign leiomyoma, which is biologically quasi-malignant due to its insidious intravascular growth [3, 9]. IVL is divided into four stages according to its extent of involvement [10]. The corresponding symptoms and signs of four stages are illustrated as follows [5, 10–12]: (1) Stage I—the lesions localize in the abdominopelvic cavity, including thrombus confined to the veins within the uterine myometrium and extending to the iliac vein or gonadal vein/renal vein from the uterine vein but not into the IVC: abdominal or pelvic masses (possibly asymptomatic), pelvic pain, abdominal discomfort, and abnormal uterine bleeding; (2) Stage II—thrombus extend to the IVC but not reaching the RA: intravenous space-occupying lesion mimicking thrombosis, lower extremity edema, bloating, and BCS in extreme cases; (3) Stage III—the lesions extend into the RA or right ventricle but below the pulmonary valve: syncopal episodes, heart failure, and even sudden death; (4) Stage IV—the lesions above the pulmonary valve and reach the pulmonary artery: chest distress, dyspnea, chest pain, non-thrombotic pulmonary embolism, and pulmonary metastatic nodules. BCS is a heterogeneous group of hepatic vascular disorders caused by hepatic venous outflow obstruction [7]. Mostly, hepatic venous outflow obstruction occurs due to underlying prothrombotic disorders and external compression by a benign or malignant lesion [13, 14]. Its clinical symptoms and signs depend on the position of occlusion and area of the liver involvement [14]. If the obstruction occurs in hepatic veins, patients usually have a rapid course presenting as abdominal pain, hepatomegaly, jaundice, and portal hypertensive bleed; if the obstruction occurs in IVC, the clinical course is insidious and patients present as lower extremity edema, superficial abdominal wall collaterals, and infertility [13, 14]. Generally, ascites, hepatomegaly, and abdominal pain are the three characteristic symptoms of BCS [15].

IVL can be anatomically divided into the lower, middle, and upper segments [16]. Upper segment of the IVC runs from the entrance of the hepatic vein to the RA [17]. IVL with extension into the upper segment could cause hepatic veins occlusion, which leads to secondary BCS [16]. IVL presenting as BCS is an exceptionally rare condition. We performed a literature search through the Cochrane library, PUBMED, and EMBASE up to September 2022. The following search terms were used: "leiomyomatosis", “intravenous leiomyoma”, “intravenous leiomyomatosis”, "Budd–Chiari Syndrome", “obstruction of the hepatic venous flow”, and “hepatic venous outflow obstruction”. Only three articles published in English were identified. The detailed information about clinicopathological features, management, and follow-up of these four cases (including our case) were summarized and described in Table 1. The mean onset age of these four patients was 54.8 years. Consistent with the literature, all but one (Patient No. 2) had a history of myoma, myomectomy, or hysterectomy. Abdominal pain, bloating or increasing abdominal circumference, and bilateral lower extremity edema were common symptoms. Only Patient No. 4 (reported in our case) exhibited dilated tortuous veins in the lower abdomen and lower back. CTA showed hepatic parenchyma congestion with nodule and septation enhancement, which was consistent with the literature [14].

**Table 1:** Summary of intravenous leiomyomatosis presenting as Budd–Chiari syndrome

Authors		Patient no.	Age (year)	Initial symptoms	Relevant medical history	Tumor lesion extension	Stage	Symptoms and signs of BCS	Involvement of hepatic vessels	Preoperative liver function	Management	Postoperative anticoagulation	Follow-up
Park SY [7]		1	Early 50 s	Abdominal pain and distention	A 15-year history of uterine myoma without any treatment	Pelvic mass, IV, IVC, RA, and RV	III	Increased abdominal birth; Pitting edema on both legs; ascites; right pleural effusion; hepatomegaly	The intracanal tumor was located in the orifice of the left and middle hepatic veins, causing obstruction	Normal, but ALB-2.69 g/dL	A one-stage surgery (TAHBSO + debulking surgery of intracardiac and intracanal tumor)	No	A pelvic mass recurred 4 months postoperatively; alive with recurrent tumor for 30 months
Barksdale J [6]		2	44	Increasing lethargy, abdominal pain, bilateral lower extremity edema, and increasing abdominal girth	Without history of myoma and hysterectomy	A mass of uterine body, right gonadal vein, IV, IVC, RA, and the right pulmonary artery	IV	Increased abdominal birth; edema on both legs; right pleural effusion; perihepatic ascites; hepatic congestion	Bland thrombus within all three hepatic veins	AST-79 IU/L ALT-104 IU/L ALP-274 IU/L. ALB-2.5 g/dL	A two-staged operation (complete tumor resection + TAHBSO)	No	No evidence of recurrence for 3 months
Kuenen BC [8]		3	76	A swollen left leg	Abdominal subtotal hysterectomy 47 years ago for unknown reasons; laparotomy surgical resection of the cervix remnant for IVL	The left common IV, IVC, and RA	III	Slightly swelling abdomen; hepatomegaly; edema on both legs; ascites	The occlusion of the large hepatic veins	Progressive elevation of AST, ALT, and LDH	Intravenous heparin and oral anticoagulants	/	Died during the third to fourth week after admission
/		4	49	Bilateral leg edema, abdominal distension	Hysterectomy for a broad ligament myoma; myomectomy of the bladder surrounding tissues	Pelvic mass, IV, IVC, and RA	III	Pitting edema over both legs; hepatomegaly; varicose veins on the abdominal wall and the right waist back	The occlusion of the hepatic venous reflux	TBIL-2.39 mg/dl; DBIL-1.14 mg/dl; TBA- 29.1 μmol/L; GGT-136U/L; ALT, AST, ALP, and ALB are normal	A one-stage surgery (BSO + incomplete resection of intracardiac and intracanal tumor)	Yes, warfarin and aspirin	Alive with residual tumor for 11 years

IVL, intravenous leiomyomatosis; IV, iliac vein; IVC, inferior vena cava; RA, right atrial; RV, right ventricle; BCS, Budd–Chiari syndrome; AST, aspartate aminotransferase; ALT, alanine aminotransferase; ALP, alkaline phosphatase. ALB, albumin; TBIL, total bilirubin; DBIL, direct bilirubin; TBA, total bile acid; GGT, gamma-glutamyltransferase; TAH, total abdominal hysterectomy; BSO, bilateral salpingo-oophorectomy

The establishment of clinical diagnoses of IVL and BCS mainly depends on the clinical presentations and imaging. Imaging examinations not only effectively facilitate the clinical diagnosis of IVL and BCS, but facilitate evaluation of the effects of treatment [18]. Ultrasonography, CT (with vascular reconstruction), and magnetic resonance imaging can determine the site, size, growth pathway, extent of invasion, and the relationship between the tumor and the surroundings [18, 19]. These imaging modalities also aid in the diagnosis of BCS by delineating the venous anatomy [14]. Echocardiography is commonly used to identify intracardiac lesions [19]. If a patient presents with typical symptoms of BCS, pelvic, intravenous, and/or intracardiac lesions are found on imaging, clinicians should raise a high suspicion of BCS caused by IVL, especially in a patient with a prior history of leiomyoma, myomectomy, or hysterectomy. A timely and proper diagnosis is important in establishing an optimal surgical plan and improving prognosis.

In light of the etiology of BCS secondary to IVL, surgical intervention to alleviate the hepatic veins outflow obstruction is the most important treatment. Although Patient No. 3 had been diagnosed with IVL of the cervix remnant, her clinical picture was interpreted as deep venous thrombosis and anticoagulation therapy was administrated [8]. During the third week after admission, her general condition declined dramatically, and she ultimately succumbed to her disease. However, the other three patients all survived owing to timely surgical tumor resection. This highlights the necessity of tumor resection as the sole curative treatment for BCS caused by IVL, particularly to alleviate hepatic vein outflow obstruction. Considering that IVL is prone to recur and is an estrogen-dependent tumor, complete tumor resection with bilateral salpingo-oophorectomy (BSO) has the lowest recurrence rate and is recommended [20]. However, only Patient No. 2 underwent complete tumor resection and TAHBSO and did not experience recurrence [6]. Both Patients No.1 [7] and No. 4 recurred in a short period after incomplete tumor resection even though they had their internal genitals removed. Liang et al. [20] concluded that complete resection with BSO had the lowest recurrence rate, incomplete resection with BSO had an intermediate recurrence rate, while incomplete resection with ovarian preservation had the highest recurrence rate.

Our case exemplifies that IVL can present as BCS, and to the best of our knowledge, it is the fourth case of IVL complicated by BCS. Combined with the literature review, we summarized the clinical characteristics, treatment, and prognosis of secondary BCS caused by IVL. Moreover, we emphasized an important characteristic of leiomyomas, which despite being benign can often occur outside of the uterus through mechanisms not fully understood. BCS caused by IVL is rare, with only few literature reports. Insufficient attention is given to this condition due to its rarity. Our case fills the current gap in this field, hoping to improve cognition of BCS and IVL. BCS caused by IVL should be considered when IVC and right atrium lesions occur in a patient with characteristics of BCS and a history of uterine myoma or hysterectomy. Most importantly, surgical resection is the only curative treatment and should be performed promptly, supported by multidisciplinary collaboration, thorough preoperative evaluation and preparation, and meticulous perioperative management.

## Conclusion

IVL can lead to secondary BCS, of which fatal complications may be avoided if handled properly and timely. BCS caused by IVL should be considered when IVC and RA lesions occur in a patient with characteristics of BCS and a history of uterine myoma or hysterectomy. Complete tumor resection with TAHBSO is the only curative treatment and should be performed as soon as possible.

## Data Availability

All data generated or analyzed during this study are included in this published article.

## Abbreviations

BCS: Budd–Chiari syndrome
IVL: Intravenous leiomyomatosis
IVC: Inferior vena cava
TAH: Total abdominal hysterectomy
CTA: Computed tomography angiography
RA: Right atrium
TBIL: Total bilirubin
DBIL: Direct bilirubin
TBA: Total bile acid
GGT: Gamma-glutamyltransferase
ALT: Alanine aminotransferase
ALB: Albumin
AST: Aspartate aminotransferase
ALP: Alkaline phosphatase
CA: Carbohydrate antigen
BSO: Salpingo-oophorectomy
